# Supplementary material for: Transcriptional Expression of Nitrogen Metabolism Genes and Primary Metabolic Variations in Rice Affected by Different Water Status
Source: Plants (Basel). 2023 Apr 14;12(8):1649. doi: 10.3390/plants12081649 (PMC10140879; doi:10.3390/plants12081649)
Supplement: Supplementary file 1 [file plants-12-01649-s001.zip › plants-2321391-supplementary.pdf]

Table S1. Selected genes for this study and primer sequences used for qRT-PCR

| Metabolism | Gene | | Gene ID | Forward (5'→3') | Reverse (5'→3') | Tm |
|---|---|---|---|---|---|---|
| Ammonium transporter | Ammonium transporter 1;1 | AMT1;1 | Os04g0509600 | ACGTCATCCAGATCCTGGTC | AGACTTGTCGTGCTCGTCCT | 59 |
| | Ammonium transporter 1;2 | AMT1;2 | Os02g0620600 | CGGCTTCGACTACAGCTTCT | GACCAGATCCAGTGGGACAC | 59 |
| Nitrate transporter | Nitrate transporter 2.1 | NRT2.1 | Os02g0112100 | AGAAGGGTGACGTCAACAGG | AGGTCGAAGCGATCGTAGAA | 59 |
| | Nitrate transporter 2.3a | NRT2.3a | Os01g0704100-02 | CGTGATGGTGCTCTTCTCCT | TGTACTTGATCCCGGTCTCC | 50 |
| | Nitrate transporter 1/peptide transporter family 2.4 | NPF2.4 | Os03g0687000 | CGAGACGTTCGAGAAGCTG | GTAGGCGTCGGAGAGGAAG | 59 |
| | Nitrate transporter 1/peptide transporter family 6.5 | NPF6.5 | Os10g0554200 | CTACCTCACCATCGCCATCT | GGTAGAGCCCCAGGTAGAGC | 50 |
| | Nitrate transporter 1/peptide transporter family 8.20 | NPF8.20 | Os06g0706400 | CAGGTGGAGGAGCTCAAGAT | ACGATCGTGGTGATGATCTG | 59 |
| Nitrogen reductase | Nitrate reductase 2 | NR2 | Os02t0770800-01 | AGGGTAGAGGTGACCCTGGA | GCTGGGTGTTGAGGGACTC | 56 |
| | Nitrite reductase | NiR | Os01t0357100-01 | ATCAACGACCTCGCGTACAT | GAGAACGGCCTTGCACAC | 56 |
| Glutamine synthetase | Glutamine synthetase 1;1 | GS1;1 | Os02t0735200-01 | CTGTGGTATCGGTGCTGACA | ATGACCTCGCCGTTGATTC | 56 |
| | Glutamine synthetase 1;2 | GS1;2 | Os03g0223400 | ACGGAGAAGGAGGGCAAG | CCACAGCAGCGTGGTCTC | 59 |
| Glutamate synthetase | Nicotinamide adenine dinucleotide-dependent glutamate synthetase 1 | NADH-GOGAT1 | Os01t0681900-01 | GGCCTGGTCGATTTTATGTG | GTGCCTTCAATGCTTCATCA | 53 |
| | Ferredoxin-dependent glutamate synthetase glutamate synthetase | Fd-GOGAT | Os07t0658400-02 | ACCCTATCAAGTCCTGTGCTT | AGCAGCATCAGCTTCATCAC | 56 |
